# Perturbing the Ubiquitin Pathway Reveals How Mitosis Is Hijacked to Denucleate and Regulate Cell Proliferation and Differentiation *In Vivo*

**DOI:** 10.1371/journal.pone.0013331

**Published:** 2010-10-20

**Authors:** Andrea Caceres, Fu Shang, Eric Wawrousek, Qing Liu, Orna Avidan, Ales Cvekl, Ying Yang, Aydin Haririnia, Andrew Storaska, David Fushman, Jer Kuszak, Edward Dudek, Donald Smith, Allen Taylor

**Affiliations:** 1 Laboratory for Nutrition and Vision Research, U.S. Department of Agriculture Human Nutrition Research Center on Aging (USDA HNRCA), Tufts University, Boston, Massachusetts, United States of America; 2 Laboratory of Molecular and Developmental Biology, National Eye Institute, National Institutes of Health, Department of Health and Human Services, Bethesda, Maryland, United States of America; 3 The Departments of Ophthalmology and Visual Sciences and Genetics, Albert Einstein College of Medicine, Bronx, New York, United States of America; 4 Department of Chemistry and Biochemistry, Center for Biomolecular Structure and Organization, University of Maryland, College Park, Maryland, United States of America; 5 Departments of Ophthalmology and Pathology, Rush University Medical Center, Chicago, Illinois, United States of America; Universidade Federal do Rio de Janeiro, Brazil

## Abstract

**Background:**

The eye lens presents a unique opportunity to explore roles for specific molecules in cell proliferation, differentiation and development because cells remain in place throughout life and, like red blood cells and keratinocytes, they go through the most extreme differentiation, including removal of nuclei and cessation of protein synthesis. Ubiquitination controls many critical cellular processes, most of which require specific lysines on ubiquitin (**Ub**). Of the 7 lysines (**K**) least is known about effects of modification of K6.

**Methodology and Principal Findings:**

We replaced K6 with tryptophan (**W**) because K6 is the most readily modified K and W is the most structurally similar residue to biotin. The backbone of K6W-Ub is indistinguishable from that of Wt-Ub. K6W-Ub is effectively conjugated and deconjugated but the conjugates are not degraded via the ubiquitin proteasome pathways (**UPP**). Expression of K6W-ubiquitin in the lens and lens cells results in accumulation of intracellular aggregates and also slows cell proliferation and the differentiation program, including expression of lens specific proteins, differentiation of epithelial cells into fibers, achieving proper fiber cell morphology, and removal of nuclei. The latter is critical for transparency, but the mechanism by which cell nuclei are removed has remained an age old enigma. This was also solved by expressing K6W-Ub. p27^kip^, a UPP substrate accumulates in lenses which express K6W-Ub. This precludes phosphorylation of nuclear lamin by the mitotic kinase, a prerequisite for disassembly of the nuclear membrane. Thus the nucleus remains intact and DNAseIIβ neither gains entry to the nucleus nor degrades the DNA. These results could not be obtained using chemical proteasome inhibitors that cannot be directed to specific tissues.

**Conclusions and Significance:**

K6W-Ub provides a novel, genetic means to study functions of the UPP because it can be targeted to specific cells and tissues. A fully functional UPP is required to execute most stages of lens differentiation, specifically removal of cell nuclei. In the absence of a functional UPP, small aggregate prone, cataractous lenses are formed.

## Introduction

Eye lens organogenesis begins with proliferation of surface ectoderm into lens epithelial cells [1], [2]. This is followed by synthesis of major lens gene products, the crystallins. Continued differentiation of epithelial cells into fibers, including packing of the fibers and intracellular removal of their nuclei results in the clear lens[3]. Red blood cells and keratinocytes also loose their nuclei [4], [5]. Whereas mechanisms for removal of cell nuclei are known for blood cells and keratinocytes, the mechanism of lens cell denucleation has remained elusive for over a century. Because cell turnover is almost non existent and expression of target genes can be directed to the lens without damage to other critical organs, this tissue presents unique opportunities to explore roles for specific molecules in cell proliferation, differentiation and development. Further, the cells and their structural molecules remain in place, in order of the sequence in which they were formed, throughout life. Because of this spatial alignment, abnormalities in developmental processes or in clearance of damaged, particularly insoluble proteins, are often observed *in vivo* as localized opacities or cataracts.

Proper cellular function is dependent upon balancing and maintenance of the proteome. Such proteostasis often engages the ubiquitin proteasome pathway (UPP) [6]–[12]. In the UPP, ubiquitin (Ub), an 8 kDa protein with 7 lysines, is linked or conjugated to substrates. Roles for most lysines on Ub have been defined. K63 is utilized during DNA repair processes, protein trafficking and inflammation [13]. K48 is required to form polyubiquitin chains and high mass species which are recognized by the 26S proteasome for degradation. K33 and K27 function in stress responses [14]. K29 on Ub aids in ubiquitin fusion degradation [14], [15] and K11 is employed in degradation of APC/C substrates [13] or eliciting ERAD responses [16]–[18]. Surprisingly, although K6 is the most readily chemically modified lysine in the Ub molecule [19], knowledge about biological requirements for K6 is very limited [20].

In this work we established for the first time that K6W-Ub has an indistinguishable structure from Wt-Ub. Expression of K6W-Ub provides an unequivocal genetic opportunity to explore roles for ubiquitination and UPP-dependent proteolysis in a biological context. In order to determine roles for ubiquitination in lens development, we expressed K6W-Ub starting at embryonic day 10.5 using a lens specific promoter. This is shortly after formation of the lens vesicle, before most of the epithelial cells have been produced and well before the formation of lens fibers. Under these conditions normal Ub remains available. We monitored incorporation of k6W-Ub into Ub-protein conjugates, deubiquitination, protein aggregation, cell proliferation, and differentiation, including lens-specific-crystallin expression, proper fiber formation, denucleation and lens clarity. Expressing K6W-Ub at higher levels, without silencing the other multiple Ub genes, delays cell proliferation. Novelly, differentiation is also delayed as indicated by delayed synthesis of the lens specific crystallins. Additional evidence of delayed differentiation is failure to: form full length lens fiber cells, reverse fiber cell alignment, lengthen the cell nucleus and remove lens fiber cell nuclei. These are required to form a clear lens. Lenses in which K6W-Ub was expressed at high levels were smaller and cataractous.

The present data allow the first unified hypothesis for lens cell removal of nuclei [21], [22]. The removal sequence starts with events that are common to mitosis and involve regulation by the UPP. First we observe degradation and/or dilution of the Cdk inhibitor p27^Kip^, a UPP degradation substrate. The Cdk1/cycB complex is activated allowing for phosphorylation of lamin. This results in disassembly of the nuclear lamin on the nuclear envelope and allows entry of DNAse IIβ into the nucleus to degrade DNA, at least in part. Expression of higher levels of K6W-Ub prevents degradation of p27^ Kip^, and thus arrests this pathway. Lamin remains intact and DNAse IIβ remains at the periphery of the nucleus.

Similar to aged lens tissue, we also found accumulation of Ub-containing moieties, particularly in insoluble fractions, in lenses in which K6W-Ub was expressed at higher levels [8]. The accumulation of proteins indicates that their rate of production exceeds the rates of removal. Together the impaired protein surveillance and aberrant development that are induced by K6W-Ub-related compromise to the UPP elucidate why the lenses become opaque, and they inform about critical functions of adequate proteolytic capacity to maintain lens function.

## Results

### Effects of K6W-mutation on structure and functions of ubiquitin

**Figure 1:**
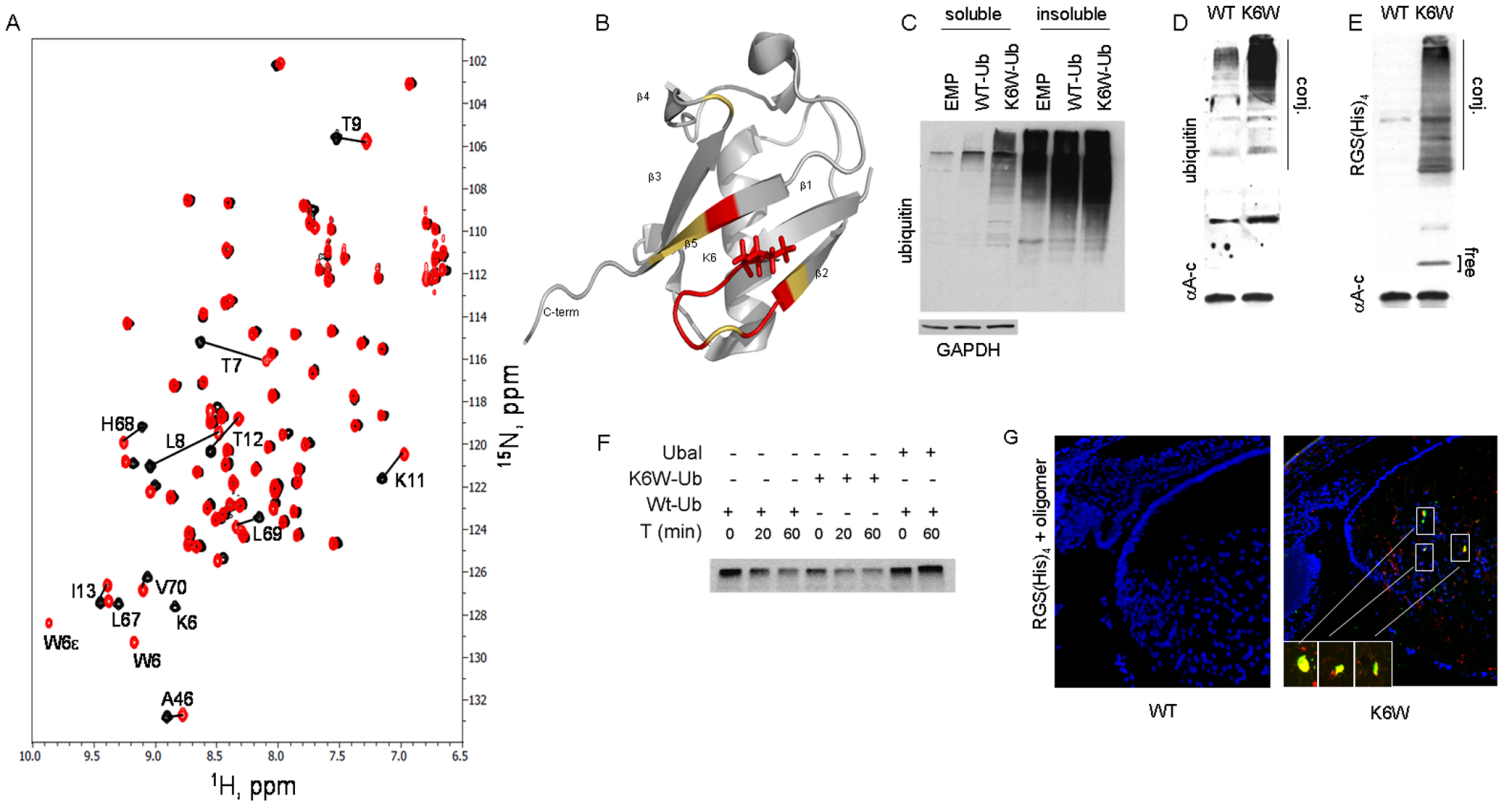
Effects of K6W-mutation on structure and functions of ubiquitin. (A) Overlay of 1H-15N HSQC spectra of Wt-Ub (black) and K6W (red). Residues showing the biggest differences are indicated. (B) Ribbon diagram of the three-dimensional structure of Wt-Ub with residues showing the largest chemical shift differences between Wt and K6W colored orange (CSP 0.1–0.2 ppm) and red (CSP >0.2 ppm). The side chain of K6 is shown in stick representation. (C) Expression of K6W-Ub in HLEC results in accumulation of high mass conjugates, especially in the insoluble fraction. Wt and K6W-Ub were expressed in HLEC via adenoviral vectors, and levels of total Ub conjugates in the cells were determined by western blotting using ubiquitin antibody. (D, E) Expression of K6W-Ub in mouse lenses in vivo using the crystallin DCR1 with 1.9 kB Cryaa promoter results in elevated levels of total Ub conjugates (D) and MRGS(His)6K6W-Ub conjugates as well as some free MRGS(His)6K6W-Ub only in transgenic lenses (E). (n = 10). Lenses from Wt and transgenic animals were lysed and endogenous levels of Ub and expression of transgene were determined by western blotting using anti-Ub or anti-RGS(His)4 respectively. (F) Deubiquitination assay. Conjugates formed with K6W-Ub are dismantled as readily as conjugates formed with Wt-Ub. Wt and K6W-Ub were labeled with ^125^I, and Ub conjugates were formed in proteasome-free fraction II of rabbit reticulocyte. Deubiquitination by isopeptidases of the ^125^I-labeled ubiquitin conjugates was determined in the presence of a 20-fold excess of unlabeled Wt-Ub conjugates. As a control, ubiquitin aldehyde (Ubal) was added to inhibit isopeptidases. (G) Immunofluorescence micrographs of P2 K6W-Ub lenses show accumulation of protein aggregates (green) that sometimes colocalize (yellow) with K6W-Ub (red). Immunohistochemistry was used to detect K6W-Ub and protein aggregates, anti-RGS(His)4 and anti-oligomer antibodies respectively. DAPI was used to stain nuclei.

We expressed and purified Ub in which K6 was replaced with W. In terms of structure and charge, this modification is analogous to biotinylation. All other lysine sites are available. Nuclear magnetic resonance (NMR) spectroscopy indicates that the overall structure of K6W-Ub is essentially indistinguishable from that of Wt-Ub, with resonances for the majority of backbone amides remaining unperturbed in the mutant Ub (Figure 1A, Supplementary Figure S1A). K6 is in a beta strand adjacent to the hydrophobic patch (formed by L8, I44, H68, V70) on Ub's surface that is recognized by various Ub-receptors. In covalently linked (i.e. K48-linked) polymers K6 is found at the junction of the hydrophobic surface in polyubiquitin chains that signal a proteolytic fate for the substrate protein (Figure 1B). K6 is observed in E2-Ub interfaces [23]. The similar structure of K6W-Ub to Wt-Ub is corroborated by its facile incorporation into high mass Ub conjugates when it is expressed in human cells (Figure 1C) and mouse tissues (Figure 1D, E). Despite these biophysical similarities with Wt-Ub and the facile incorporation of K6W-Ub into conjugates in cell free assays, these conjugates are not degraded effectively *in vitro and in vivo* [19].

Roles for K6W-Ub were pursued in human lens epithelial cells (HLEC) in culture and *in vivo*. Overexpression of Wt-Ub in HLEC results in 4-fold and 1.2 fold enhanced levels of high mass conjugates in the soluble and insoluble fractions, respectively, indicating that HLEC, like many other types of cells in culture, are limited with respect to supplies of free Ub (Figure 1C) [19] [24],[25], [26]. In comparison, expression of K6W-Ub results in >16-fold and >1.2-fold greater accumulation of conjugates in the soluble and insoluble fractions, respectively, including significant levels of moieties which are large enough to remain in the stacking gel (Figure 1C). The extensive accumulation of high mass conjugates in lenses in which K6W-Ub is expressed (Figure 1D, E) corroborates in an *in vivo* setting that K6W-Ub is conjugation competent but conjugates which incorporate this variant are proteolytically resistant [19]. Importantly, the accumulation of K6W-Ub-containing conjugates is not due to differences in rates of deubiquitination (Figure 1F) since conjugates formed with K6W-Ub are dismantled as readily as conjugates formed with Wt-Ub (Figure 1F, Supplementary Figure S1E). Taken together, these data imply that K6W-Ub is an effective competitive inhibitor for the UPP the expression of which can be targeted to desired cells and tissues.

### Effects of K6W-Ub-induced UPP inhibition on lens cell proliferation, differentiation, lens formation and clarity, and proteostasis

**Figure 2:**
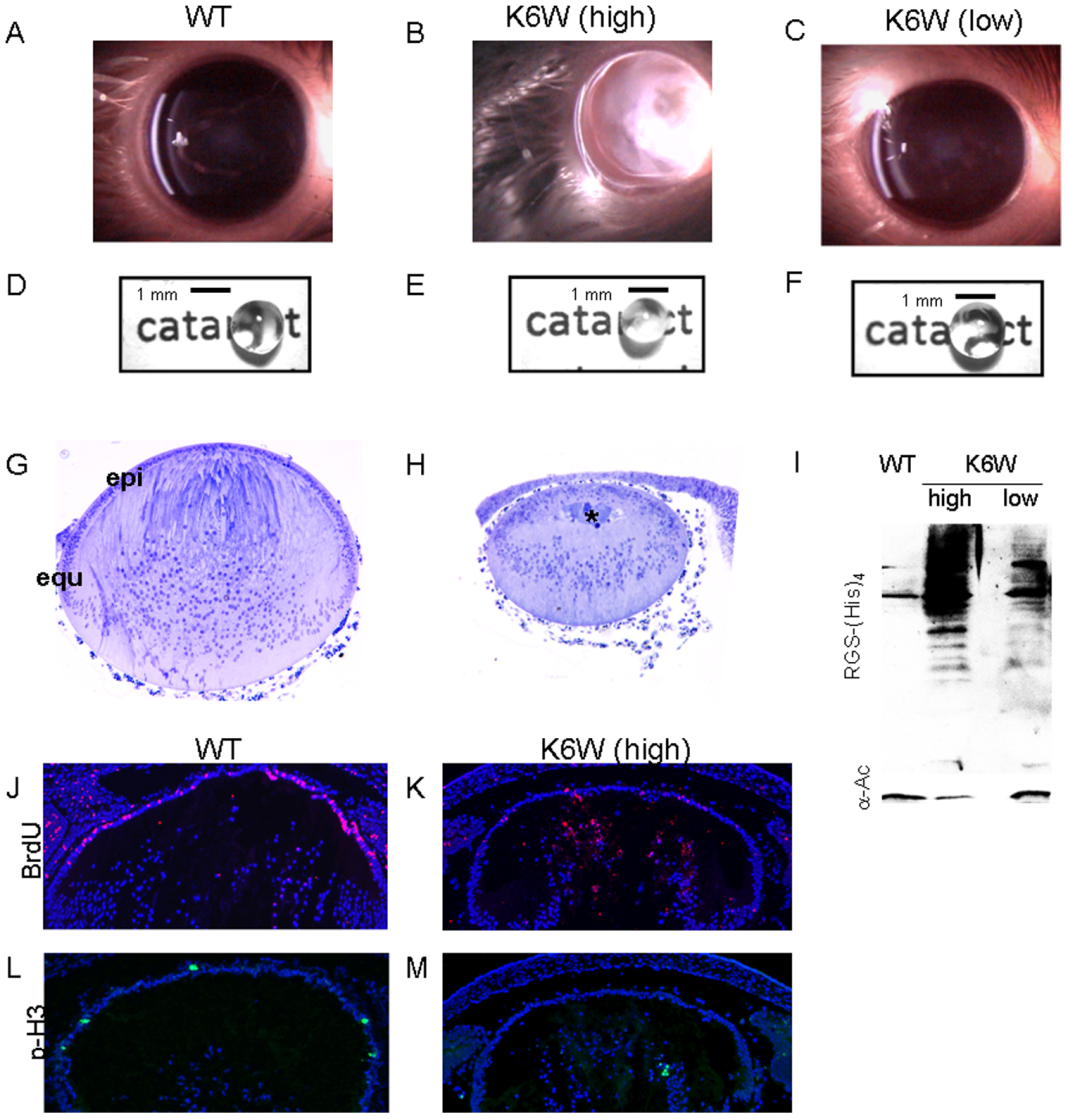
K6 on Ub is essential for proper lens formation and clarity. Slit-lamp photographs of P90 mouse lenses. Lenses expressing high levels of K6W-Ub (A) show severe cataracts whereas lenses from animals expressing low levels of K6W-Ub (C) are clear, comparable to wild type (B). (D, E, F) Head-on photographs of P30 lenses. Lenses expressing high levels of K6W-Ub are cloudy and opaque. Note that the print behind the lens in panel E cannot be seen as it is in panels D and F. Lens from animals expressing low levels of K6W-Ub are clear comparable to Wt. (G, H) Light micrographs from E18.5 days Wt and K6W-Ub lenses. Lenses expressing high levels of K6W-Ub were ∼2/3 the size of Wt lenses. (I) P30 lenses from high or low K6W-Ub-expressing animals show different levels of K6W-Ub-containing conjugates. Lenses from Wt and transgenic animals were lysed and expression of transgene was determined by western blotting using anti-RGS(His)4. (J–M) Fluorescent micrographs of E18.5 K6W-Ub-expressing lens show attenuated proliferation compared to Wt. (J, K) BrdU (red) incorporation assay was used to detect S-phase cells in mouse lenses. K6W-Ub-expressing lenses show limited incorporation of BrdU compared to Wt. (L, M) Phospho-H3 (green), also shows that K6W-Ub-expressing lenses have decreased proliferation compared to Wt lenses. Immunohistochemistry was used to detect incorporation of BrdU and expression of phospho-H3, using anti-BrdU and anti-phospho-H3 antibodies respectively. DAPI was used to stain nuclei.

It is not possible to target proteasome inhibitors to the lens without affecting most other organs. To overcome this barrier multiple transgenic K6W-Ub lines were generated in which the Ub variant was expressed at high and low levels in the lens. The lens was chosen because a) metabolism in lens epithelia is comparable to that of cells in many other tissues, b) to avoid embryonic lethal effects that might affect critical organs, c) aberrations in the proteome might lead to readily observed opacity, and d) because patterns of cell proliferation and differentiation are well documented and readily observed. Mice which expressed higher levels of K6W-Ub showed severe opacities or cataract and had higher levels of high mass Ub conjugates (Figure 2B, E, I), whereas lenses from animals which expressed lower levels of K6W-Ub (Figure 2C, F) or overexpressed Wt-Ub were clear (Supplementary Figure S1B) comparable to non- transgenic animals (Figure 2A, D). They also accumulated lower levels of high mass conjugates (Figure 2I).

In some cells insolubilization may be conceived as a means to limit toxicity of abnormal proteins. However, in the lens such precipitation results in opacification, hence tissue dysfunction. Interestingly, lenses with high levels of K6W-Ub show higher levels of oligomerized proteins (green) which colocalize with K6W-Ub (red), corroborating the formation of conjugates and deposition of K6W-Ub in function-compromising protein aggregates (Figure 1G). Similar results, as well as enhanced actin aggregation, were observed in cell culture models (Supplementary Figure S1C, D). This indicates that like neurodegeneration diseases [10]–[12], cataracts are also etiologically related to accumulation of soluble and insoluble Ub conjugates [8], and a functional UPP with Ub intact is required to elicit proper protein clearance. These data are consistent with prior observations that cells in which K6W-Ub is expressed at high levels are stress-sensitive [19], [24].

Critical to successful organogenesis is proper initiation and regulation of cell proliferation. E18.5 lenses which expressed high levels of K6W-Ub showed limited proliferation in the epithelial layer (Figure 2J, I vs. K, M). In Wt lenses proliferation, as indicated by the S-phase indicator BrdU, is observed throughout the epithelial layer (Figure 2J, pink). In contrast, far lower numbers of S-phase cells were found in the anterior epithelial layer of K6W-Ub-expressing lenses (Figure 2K). Instead, BrdU incorporation was present in the anterior fiber mass area, where it is usually not observed. Thus, although they begin to transform to fibers, some K6W-Ub expressing fiber cells have not completed withdrawal from the cell cycle, and the normal differentiation program is delayed. The proliferation data was corroborated by examining phosphorylated histone H3 (p-H3), an indicator of G2/M phase. Whereas, Wt lenses show anterior epithelial cells positive for p-H3 (Figure 2L, green) this was not observed in K6W-Ub-expressing lenses (Figure 2M). Instead, some fiber cells in K6W-Ub lenses are positive for p-H3 (Figure 2M) demonstrating once more that these cells are in cycle and not yet fully differentiated.

Corroborating the limited proliferation and delayed differentiation in lenses in which K6W-Ub is expressed, such lenses were about 2/3 the weight and approximately 75% the volume of Wt lenses or lenses in which the K6W-Ub was expressed at lower levels (Figure 2D–F). In addition, lenses that expressed K6W-Ub are relatively flat across the anterior surface and more rounded at the posterior, while for Wt it is the opposite (Figure 2G, H).

**Figure 3:**
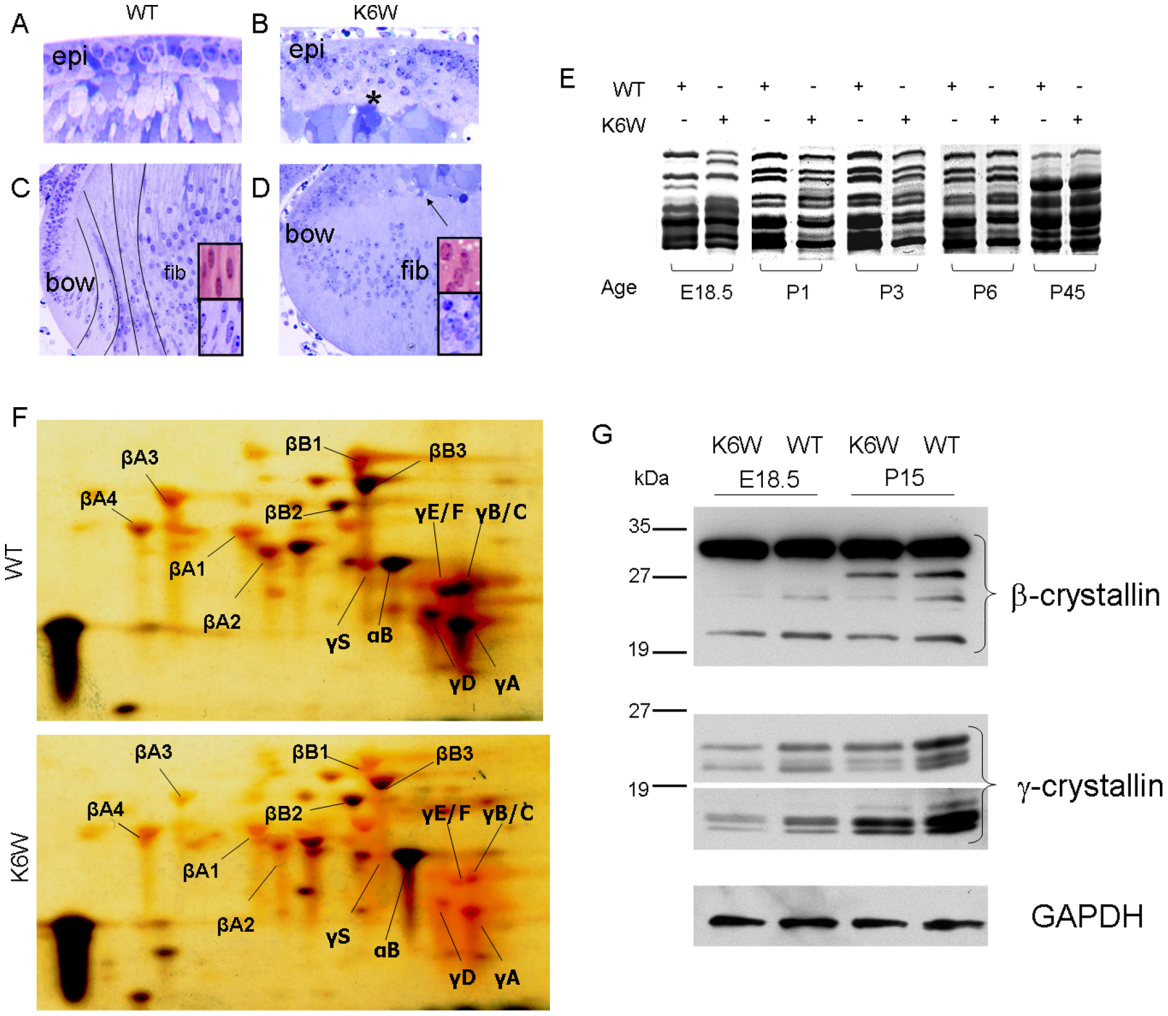
K6 on Ub is required to direct lens proliferation and differentiation and denucleation. (A–D) Light micrographs of epithelium (epi), bow region and nuclei from E18.5 days Wt and K6W-Ub-expressing lenses. The epithelium of lenses expressing K6W-Ub (B) is profoundly altered compared to the single layered epithelium of Wt lens (A). The transgenic lens epithelium shows multiple layers of cells and accumulation of cellular debris and vacuoles interposed between the anterior tips of the fibers and the epithelium (asterisk). Compared to Wt lenses, fibers (fib) of K6W-Ub transgenic lenses do not fully elongate or meet the anterior epithelium (arrow) (D). In addition, the concave to convex curvature found in Wt lenses (black lines) (C) is not apparent in K6W-Ub-expressing lenses. Nuclei at the bow region do not elongate in lenses expressing K6W-Ub (boxes). (C vs. D, upper boxes). (E) Gel profiles demonstrate that the complement of crystallin proteins in which K6W-Ub was expressed are different from the proteins in the Wt animal from E18.5 to P3. By P6 the protein profiles are more similar. Lenses of E18.5, P1, P3, P6 and P45 were lysed and proteins were separated by SDS gel electrophoresis. Gels were stained with Coomassie blue. (F) 2D electrophoresis of P1 Wt and K6W-Ub-expressing lenses. K6W-Ub transgenic lenses show lower expression of crystallin proteins when compared to wildtype. Lenses of P1 animals were lysed and proteins were separated by isoelectric focusing then SDS gel electrophoresis. Gels were stained with silver. Note the absence of several β- and most γ- crystallins from the transgenic lens. (G) Western blot of β- and γ- crystallins in E18.5 and P15 lenses. Levels of β- and γ- crystallins in transgenic lenses at E18.5 and P15 are lower as compared to wildtype lenses. Lenses from wildtype and transgenic animals were lysed and expression of crystallins was determined by western blotting using anti-β- and γ- crystallins antibodies. Equal loads are shown by western blotting using anti-GAPDH antibody.

**Figure 4:**
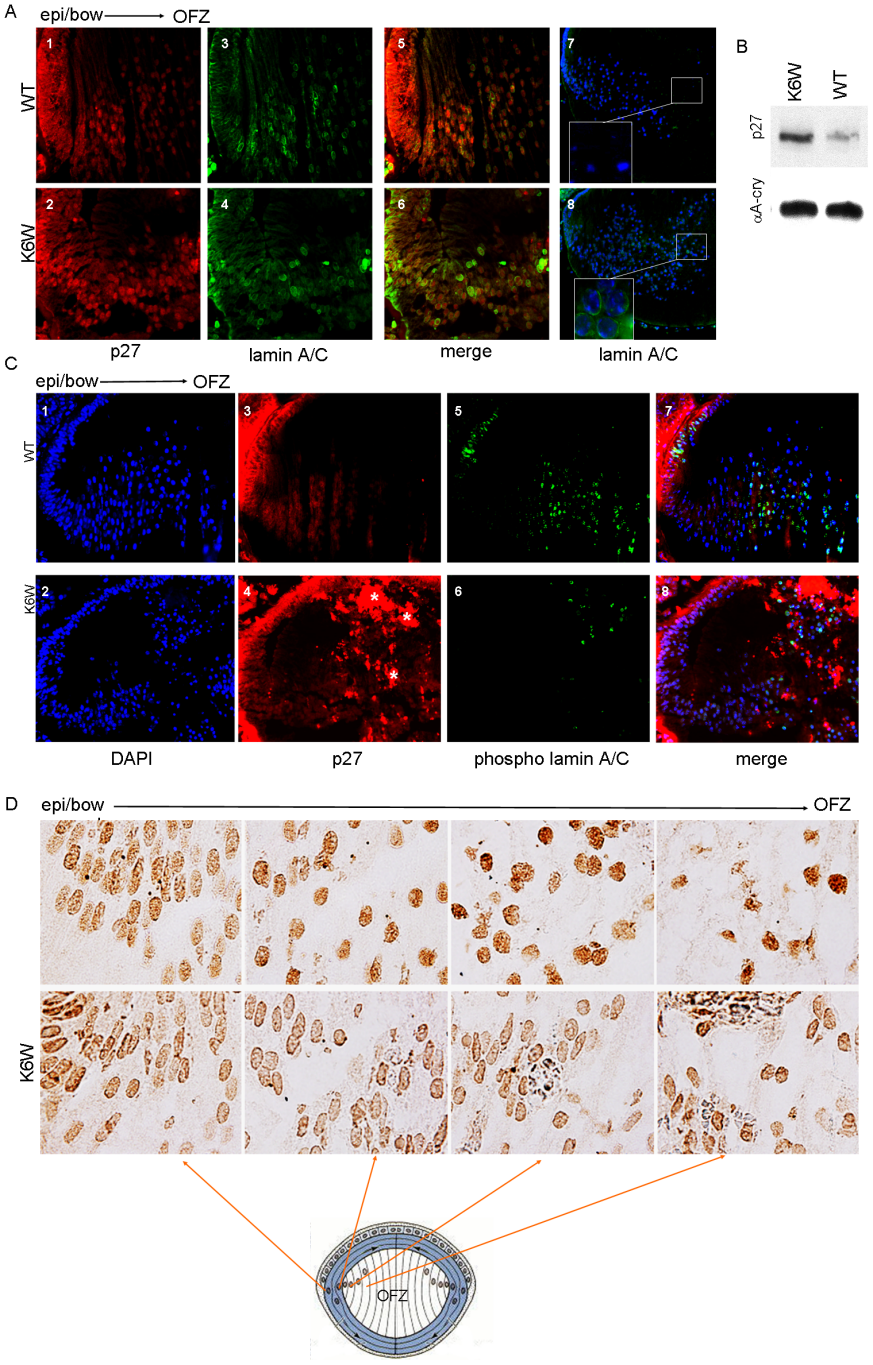
K6 on Ub is required to direct lens denucleation. (A) Fluorescent micrographs of E18.5 K6W-Ub-expressing (panel 2, 4, 6, 8) and Wt (panel 1,3, 5, 7) lenses show distribution of p27 (red) and lamin A/C (green). In Wt lenses p27 is localized at the bow region of the lens and is concentrated in nuclei, but is also present in the cytoplasmic compartments of lens fiber cells. There is a gradual decrease in p27 expression from the bow region to the OFZ. Transgenic lenses (panel 2) show greater retention of p27 (note elevated red staining) in nuclei and the fiber mass as compared to Wt (panel 1). Intact nuclear membranes in the core of the lens are only seen in transgenic lenses (panel 8). While in Wt, no nuclear membranes are apparent and only DNA fragments are observed in the core of the lens (panel 7). (B) P1 K6W-Ub transgenic lenses show stabilization of p27 protein. Lenses were lysed and levels of endogenous p27 were determined by western blotting using anti-p27 antibody. Equal loads are shown by western blotting using anti-αA crystallin antibody. (C) Fluorescent micrographs of E18.5 K6W-Ub-expressing (panel 4) and Wt (panel 3) lenses show distribution of p27 (red) and phosphorylated lamin A/C (green) (panels 6 and 5). Localization of p27 is the same as in (A, panel 1 and 2). Asterisks show non-specific staining (panel 4). In addition, lenses that express K6W-Ub (panel 6) show decreased levels of phosphorylated lamin A/C (green) toward the lens core as compared to Wt (panel 5). Immunohistochemistry was done to detect levels of p27, phosphorylated lamin A/C, lamin A/C using anti-p27, anti-lamin A/C (phospho Ser 392) and anti-lamin A/C, respectively. DAPI was used to stain nuclei. Note that phosphor-lamin is detected more in the dividing epithelia of Wt lenses rather than transgenic lenses. (D) Light micrographs of P2 mice Wt and K6W-Ub transgenic lenses show the distribution of DNAse IIβ (dark brown). Comparable sections from the bow region to the edge of the OFZ of the lens. K6W-Ub-expressing lenses show accumulation of DNAse IIβ around the nuclear envelope for all regions. Going from the bow towards the OFZ, more DNAse IIβ enters the nucleus and less accumulates at the nuclear envelope. Immunohistochemistry was done to detect distribution of DNAse IIβ using anti-DNAse IIβ antibody.

Also required to facilitate transparency is proper intracellular protein organization and intercellular interdigitation. Epithelial cells line the anterior surface of the normal lens. Vacuoles which interrupt the close packing of the protein-dense epithelial or fiber cells result in discontinuities in the index of refraction, resulting in opacification. In the Wt-Ub lens the single layer of cuboidal epithelial cells which is normally found at the anterior equator withdraw from cell cycle. Here, these cells begin to differentiate. This is coupled with the massive expression of crystallin proteins, transforming the cuboidal equatorial cells to elongated fibers at the bow, (Figure 2G, Figure 3C). The continuous addition of new fiber cells at the lens periphery leads to a gradual inward movement and compression of older cells toward the center of the lens where intracellular organelles are lost forming an organelle-free zone (OFZ, Figure 4A, C, Supplementary Figure S3A). This highly regulated process persists throughout lifetime. In the outer elongating fibers, nuclei become oval-shaped (Fig 3B, inserts). Eventually the nuclei are degraded as fibers move toward the interior of the lens which forms the visual axis (Figure 4A, 4C, 4D and Supplementary Figure S4A). Another early indicator of the differentiation process is the realignment of the fibers. Whereas at the equator the elongating fibers in the bow have a concave curvature (Figure 3C), they develop convex curvature as they elongate further and meet up with equivalent fibers from the opposing side of the lens and form sutures at the anterior and posterior tips (Figure 2G, 3C). These processes do not proceed as scheduled in the K6W-Ub-expressing lens. Instead, the epithelium is composed of multiple disorganized layers and appears thicker (compare Figure 3B vs. 3A, Supplementary Figure S2B, D vs. A,C). To corroborate the origin of these disorganized cells, connexin 43, an specific epithelial cell marker, was localized. In K6W-Ub expressing lenses the thick layer of disorganized cells is positive for connexin 43 indicating that these cells are epithelial an not degenerating fibers (Supplementary Fig S2A, 2B). Just posterior to these layers of epithelial cells, cellular debris accumulates and vacuoles form (Figure 2H asterisk, 3B asterisk, Supplementary Figure S2F vs. E). The fibers cells neither elongate fully, precluding adapting the convex shape, nor do they interdigitate with the opposing fiber partners. Fibers in the K6W-Ub-expressing lens begin to differentiate more anteriorly resulting in an incompletely formed and anterior bow (Figure 2H vs. G, Figure 3D vs. C). These fibers appear to be less tightly packed than in the lenses of the Wt littermates (Supplementary vs. E). Furthermore, nuclei remain round at the bow (compare Figure 3D vs. 3B, insets). By P2, when fibers in Wt lenses begin to denucleate, nuclei are retained in the core of the lens which express higher levels of K6W-Ub (Supplementary Fig S4A, Figure 4C). Additional indicators of K6W-Ub-induced altered differentiation are retention of ER and mitochondria as well as accumulation of aggregates and debris (Supplementary Figure S3A–D). Retained organelles and debris prohibit the formation of the clear optic fiber that is required for passage of light.

Comparison of protein expression corroborates the anatomic indications of K6W-Ub-induced problems in the differentiation program. In lens α-, β- and γ-crystallins are synthesized sequentially [27]. Synthesis of these proteins is observed in the protein profiles shown in Figure 3E–G. In comparison, there is delayed expression of some β, and most of the γ-crystallins in the K6W-Ub lens (Figure 3E–G). For example, it is clear that at E18.5 and P1 synthesis of crystallins (γ-B, γ-C γ-D, γ-E, γ-A, γ-S,) and several β-crystallins (β-B1, β-A3, β-B3, β-A1, β-A2) is markedly delayed in the lenses which express the K6W-Ub. These differences are particularly readily observed in the γ-crystallins region of the 2D gels shown in Figure 3F. The difference appears to be sustained even at P15 (Figure 3G).

Several differences from prior indications of sequences of crystallin synthesis were also indicated. Ueda et al. indicates that α-B and γS-crystallins increases upon early postnatal lens development. We see that, in equally loaded samples, γS-crystallin is present at P1 in Wt lenses as expected and it is less obvious in the K6W-Ub lens. (Figure 3F, 3G). However, in contrast with their prediction α-B is a more prominent protein in the K6W-Ub expressing lens. The data are consistent with expression of K6W-Ub resulting in abnormal differentiation after the onset of α-crystallins and prior to onset of γ-crystallin expression. The absence of a significant effect on α-crystallin expression is expected because of the construct we used to express the transgene. Earlier expression is not possible at present in this context. The delay in population of the fibers with γ-crystallins provides another clear indication that expression of the transgene impairs timely lens cell differentiation and also explains the difference in weights of the lenses. This difference diminishes with time such that after ∼P30 Wt and K6W-Ub-expressing animals have more similar protein profiles (Figure 3E). Thus, the effect of K6W-Ub is observed at early stages of lens development, when the lens continues to add most of its fiber mass. Notably, at early ages, i.e. E18.5, P1, and P3 the proportion of K6W-Ub to other proteins is higher than at P45 (Supplementary Figure S4B). The relative levels of the transgene decreased dramatically, due to dilution by elevated levels of crystallins and massive expansion of cell volume (Supplementary Figure S4B). This data was corroborated by measuring the expression levels of the transgene. By real time PCR expression of K6W-Ub relative to GAPDH does not change with age (Supplementary Figure S4C). This corroborates our hypothesis that K6W-Ub protein levels are diluted by the massive production of crystallins in the lens fibers. This reduction in the relative levels of K6W-Ub may explain the amelioration of the phenotype in K6W-Ub-expressing lenses in regions of the lens that are produced at older ages (Supplementary Figure S4A). Collectively, the data indicate that expression of K6W-Ub results in retardation and alterations in the normal lens cell proliferation and differentiation programs, thus, emphasizing that a Ub with K6 intact, is required for proper and timely progress of differentiation which results in a clear, functional lens.

### Expression of K6W-Ub reveals a pathway by which lens cells disassemble their nuclei

Nuclei must be removed to allow for unimpeded passage of light through the lens fibers. The retention of nuclei, together with observations of G2/M arrest in cells in which K6W-Ub is expressed [24], and similarly arrest in fibers in the K6W-Ub expressing lens (Figure 2M), as well as concepts that removal of nuclei might exploit mechanisms that are normally employed to dismantle cell nuclei during mitosis [21] allowed critical new insights into how fiber cells denucleate [21]. Specific to this work, we tested the novel hypothesis that a properly functional UPP, including adequate concentrations of Ub with K6 intact, is required to elicit a chain of events that results in destruction of nuclei (and other organelles), leaving an optically clear OFZ the Wt [28]–[32].

In support of this hypothesis we observe in Wt lens a spatially and developmentally coordinated dilution (due to rapid synthesis of crystallins) or/and destruction of p27 in the cytoplasmic and nuclear compartments of the lens fibers in the OFZ (Fig 4A, panels 1, 3, 5 and Figure 4B,C) as compared with epithelial or the bow regions. In the Wt lens, there is also a concerted activation of Cdk1/cyclinB [21], as indicated by phosphorylation of nuclear lamin A/C at serine 392 (same phosphorylation site as in mitosis) (Fig. 4C, panels 5, 7). In the Wt lens, phosphorylated lamin is dismantled allowing for permeabilization of the nuclear membrane. In the lens core cell nuclear membranes are no longer obvious (Figure 4A, panel 7) and DNAse IIβ, a lysosomal enzyme, can gain entry to the nucleus (Fig. 4D, note dark brown nuclear staining in the cells which will soon lose their nuclei, right panels, vs cells that have not entered the terminal differentiation process, left panel) [33], [34]. This leaves low levels of remnant DNA fragments (Figure 4A panel 7 vs. panel 8), thus, establishing the OFZ. In contrast, in the smaller, K6W-Ub-expressing lens there is delayed synthesis of crystallins (Figure 3E–G), higher concentrations of nuclear and cytoplasmic p27 (Figure 4A panels 2 and 6; Figure 4C panels 4 and 8; Figure 4B), and limited phosphorylation of lamin (Figure 4C, panel 6). Failure to phosphorylate lamin is associated with retention of intact nuclear membranes and nuclei as well as exclusion of DNAse IIβ from the fiber cell nucleus, leaving intact DNA (Figure 4A panel 8). The colocalization of p27 and lamin A/C, where nuclear membranes are intact in Wt and K6W-Ub expressing lenses is consistent with our hypothesis that Cdk-dependent phosphorylation of p27 is a prerequisite for its degradation, activation of the kinase, lamin phosphorylation and eventually for denucleation. Also note the clear phosphorylation of lamin in the proliferating epithelia of the control lens whereas this is far less obvious in the K6W-Ub expressing lens in this zone suggesting that nuclear membrane breakdown during lens denucleation is the same as in mitosis. Taken together these data identify functional Ub as essential for denucleation during terminal differentiation and to avoid cataractogenesis at early stages of organogenesis. They also imply a new function for the UPP in directing lens cell proliferation and multiple stages of differentiation.

**Figure 5:**
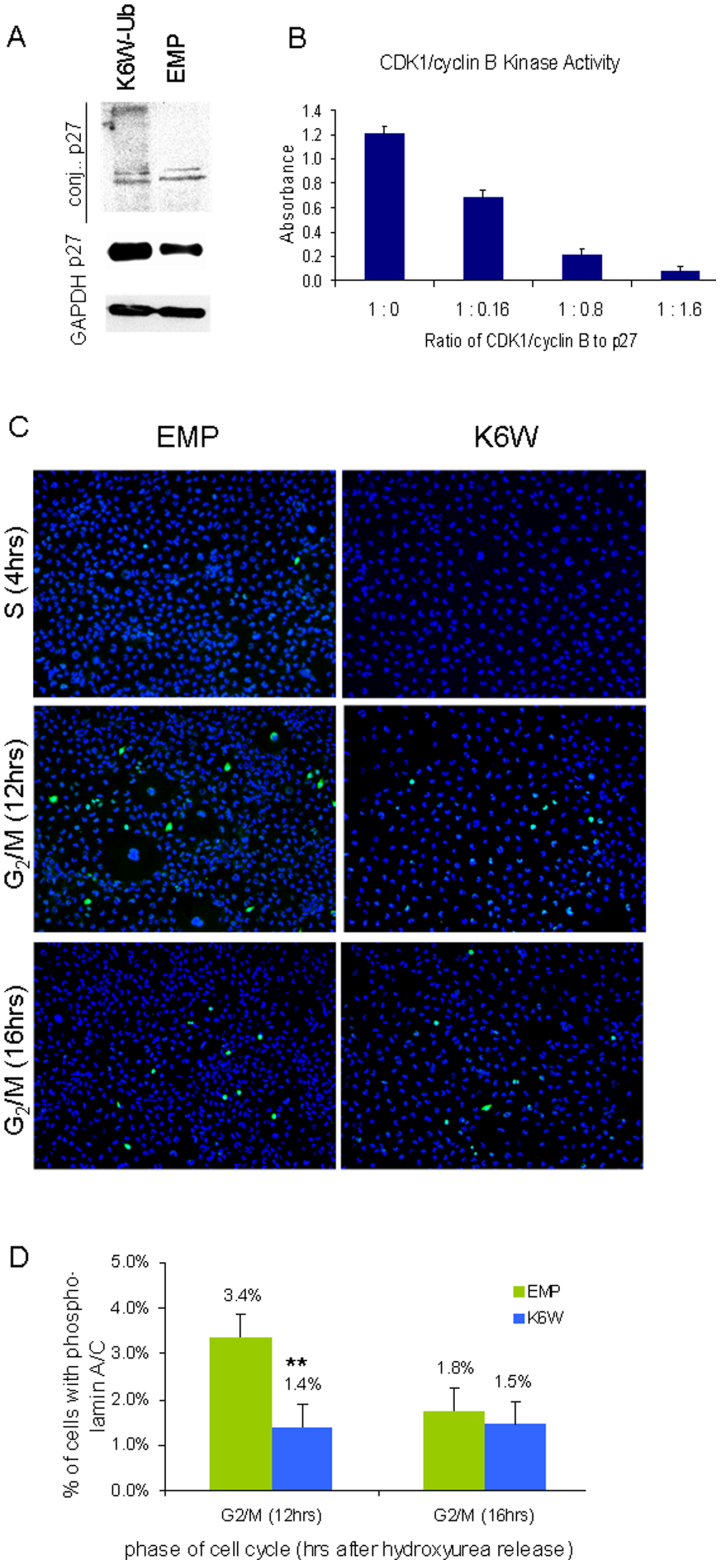
K6 on Ub is required to degrade p27. (A) HLEC expressing K6W-Ub show stabilization of p27 in G2/M synchronized cells. Cells were synchronized in early mitosis phase using nocodazole. K6W-ubiquitin was expressed in HLEC via an adenoviral vector and levels of endogenous p27 were determined by western blotting. (B) Cdk/cyclin B activity is blocked in a dose-dependent manner by the addition of p27 recombinant protein in vitro. Cdk1/cyclin B activity was determined in vitro by the quantification of phosphorylated-Rb by ELISA. (C) HLEC expressing K6W-Ub show decreased phosphorylation of lamin A/C in early and late G2/M phase. Cells were synchronized at G1 phase with hydroxyurea. After release of hydroxyurea, cells were allowed to grow for 4, 12 and 16 hrs to synchronize at S, early and late G2/M phase respectively. Immunohistochemistry was done to detect levels of phosphorylated lamin A/C using anti-lamin A/C (phospho Ser 392). (D) HLEC in which K6W-Ub was expressed show significantly lower amounts of phosphorylated lamin A/C at early and late G2/M phase. Cells positive for phosphorylated lamin A/C were counted from 10 different fields from fluorescent micrographs in panel C. Positive cells were averaged and statistics were performed at p<0.05.

Mitotic models were used to corroborate and extend the above results. p27 is seen to accumulate both at its native mass and as high mass conjugates in cells synchronized in the G2/M phase of the cell cycle when K6W-Ub is overexpressed, but not when cells are infected with control virus (Figure 5A). *In vitro* tests showed that p27 indeed inhibits the Cdk1/cyclinB complex and that its inhibition is dose dependent (Figure 5B). We then observed that for synchronized cells there are significantly higher levels of phosphorylated lamin in control-infected as compared with K6W-Ub-expressing cells (Figure 5C green, Figure 5D). This corroborates the hypothesis that to promote nuclear disassembly it is necessary at early mitosis to enhance levels of phosphorylated lamin and that that requires diminished concentrations of p27 to allow for activation of the kinase complex. Prior observation of p27-induced inhibition of Cdk1/cyclin B activity and arrest of cells at the G_2_/M transition are consistent with our data [35], suggesting that, as in other cells, in lens inhibition of Cdk1/cyclin B is a direct consequence of the accumulation of p27 (or other Cdk inhibitors) and that expression of K6W-Ub enhances this phenomenon. Thus, the data define a novel pathway by which nuclei can be removed during lens cell differentiation.

## Discussion

K6W-Ub has a structure that is indistinguishable from the Wt molecule and readily supports ubiquitination, but incorporation of Ub with mutations at K6 results in perturbed UPP function and profound abnormalities in proteostasis, differentiation, and developmental as well as in age-related protein precipitation disease phenotypes. Cataract is clearly among these. In this work we made no effort to silence the multiple endogenous Wt-Ub genes. Thus, the effects due to expressing the K6W-Ub variant are probably minimized. The data establish new roles for Wt-Ub and the UPP to execute these critical developmental functions and they allowed us to elucidate how denucleation is regulated. They also demonstrate the utility of the K6W-Ub variant to unequivocally explore roles for ubiquitination and the UPP in multiple physiologic settings.

Novelly, we propose that a specific program for destruction of the lens fiber cell nucleus is adapted from normal early mitotic events. This involves diminishing levels of p27, activating Cdk1/cyclin B, phosphorylating lamin and disassembly of the nuclear envelope. In the lens, denucleation is completed by entry of DNAse IIβ and degradation of DNA. A corollary is that having adequate concentrations of Wt-Ub is crucial for these processes to proceed. It will be fascinating to elucidate how the full mitotic program is diverted in lens cells to allow denucleation but not mitosis.

Interestingly, Ubs linked at K6 are predicted to have a closed conformation similar to that of K48-linked polyubiquitin chains that do direct protein substrates for proteolysis [36] and substrates conjugated with K6W-Ub are readily deubiquitinated. Moreover, it is clear that inter ubiquitin linkages which involve lysines other than those built upon K6 must be formed ([37]; [16]. Nevertheless, substrates which include ubiquitin in which tryptophan replaces lysine at position 6 are not bound effectively by Ub-recognition motifs at the 26S proteasome and, they retard degradation. Since with intact Ub accumulation of soluble and insoluble aggregates is avoided, the appearance of K6W-Ub along with protein precipitates indicates that having K6 intact on Ub is required for the timely degradation of proteins in this *in vivo* mammalian tissue setting. Aggregation and precipitation of proteins is particularly problematic in the lens which must remain free of insoluble aggregates to remain clear to pass light. These data are corroborated by prior demonstrations that ubiquitinated moieties accumulate in cataractous precipitates in older stressed lenses [8] and by recent data showing age- and disease- related accumulation of damaged proteins, including Ub, and dysfunction in the brain of animals in which the proteasome is mutated [38]. Furthermore, they suggest that the present results are generalizable, i.e. that having Ub with K6 intact is required for formation and function of many other tissues.

The availability of adequate amounts of Ub with K6 intact is required for proper transit through the cell cycle and to initiate the developmental processes that precede denucleation in the lens. These include elaboration of lens specific proteins, the many changes in cell structure as lens cells become fibers, the realignment of the fibers, evolved changes of nuclear shape and finally removal of the nuclei. The eventual formation of lens fibers that appear normal at the periphery of the affected lens in high expressing K6W-Ub lenses is consistent with altered expression of proteins- even in elongating epithelial cells- as the animals mature and age.

Finally, in addition to providing a new way to unequivocally establish roles for ubiquitination in physiologic processes, the data indicate that modification of Ub or discovery of molecules that result in altered Ub metabolism may be useful to alter cell proliferation such as encountered in cancer and secondary cataract. The animal model may also be adapted to test anti-cataract or anti-aging drugs.

## Materials and Methods

### Ethics Statement

All animals were generated and maintained at the National Institute of Health or at the Jean Mayer USDA Human Nutrition Research Center on Aging at Tufts University under specific pathogen-free conditions in accordance with institutional guidelines. This study was carried out and approved under the Jean Mayer USDA Human Nutrition Research Center on Aging at Tufts University IACUC protocols TA-66 and TA-67 and in accordance with the Animal Welfare Act provisions and all other animal welfare guidelines such as the NIH/Guide for the Care and Use of Laboratory Animals.

### Structural properties of polyubiquitin chains in solution [39]

All NMR studies were performed on a cryoprobe-equipped Bruker 600 MHz spectrometer at 23°C. NMR samples of WT and K6W-ubiquitin (concentrations 0.5–1.0 mM) were prepared in a buffer containing 20 mM sodium phosphate at pH 6.8, 7% D2O, and 0.02% (w/v) NaN3. Recombinant proteins were obtained using *E.coli* expression and purified as described [40]. NMR signal assignment for K6W-ubiquitin was obtained using homonuclear 2D TOCSY and NOESY experiments combined with 2D 1H-15N HSQC and 3D 15N-edited TOCSY and NOESY. NMR signal assignments for Wt-ubiquitin were used as a starting point. Perturbations in amide resonances resulting from the mutation were quantified using chemical shift perturbations (CSPs) calculated as CSP  =  [(Dd*H*)2+(Dd*N*/5)2]1/2, where Dd*H* and Dd*N* are the observed chemical shift changes for 1H and 15N, respectively.

### Gene Preparation

Ubiquitin octamer DNA constructs of both MRGS(His)_6_-Wt-Ub and MRGS(His)_6_-K6W-Ub were cloned into pCDNA3 vector (Invitrogen) and the sequences were verified. All eight ubiquitin sequences in each octamer have an MRGS(His)_6_ tag at the amino terminus. Both octamer constructs were subcloned into modified pEGFP-1 vector which comprise an EcoRI/EcoRV DCR1 promoter sequence insertion as described previously [41].

### Generation of transgenic animals

Transgenic mice were generated and maintained in FVB/N background. In these animals, expression of MRGS(His)_6_-Wt-Ub and MRGS(His)_6_-K6W-Ub is under the control of the αA-crystallin promoter. The mini genes that contain the promoter; encoding sequences and poly-adenylation sequence DNA were injected into FVB/N fertilized oocytes in AECOM Transgenic and Gene Targeting Facility and NEI Genetic Engineering Facility. The transgenic mice were identified by PCR-based genotype. Multiple lines of animals were created [42], [43].

### Deubiquitination Assay

Wt or K6W-ubiquitin was labeled with ^125^I, and ubiquitin conjugates were formed in proteasome-free fraction II of rabbit reticulocyte. Deubiquitination assay was performed as described previously [19].

### Cell Culture and Synchronization

HLEC were grown as described previously [19]. HLEC were synchronized at G_0_/G_1_ and G_2_/M in the presence of hydroxyurea and nocodazole, respectively. The G_0_/G_1_ arrested cells resumed cell cycle upon removal of the hydroxyurea. Cells were then collected or fixed after hydroxyurea release for cell extract preparation or immunohistochemistry analysis. Cells synchronized at G2/M were used to prepare lysates. Analysis of cell cycle by flow cytometry was performed as described previously [19].

### Generation of recombinant adenoviruses and infection with adenoviral vector

Adenoviruses expressing GFP along with (His)_6_-Ub or K6W-Ub or without GFP were generated as described previously [19], [24]. HLEC were arrested in G_0_/G_1_ and G_2_/M by hydroxyurea and nocodazole, respectively, and infected with K6W-ubiquitin or empty adenovirus for 24 h.

### Cell and lens extract preparation

Extracts from HLEC were prepared as described previously [8], [19]. Lenses from K6W and Wt animals were homogenized directly in SDS-PAGE loading buffer. Protein concentrations for cell extracts were determined by the Coomassie Plus Protein Assay (Pierce, Rockford, IL) and protein concentration from lens lysates was adjusted by densitometric analysis using Coomassie blue stained SDS-gels.

### Western blot analysis

Standard protocols were used for western blotting as described previously [24]. β-actin and αA-crystallin were used for normalizing the protein load. Comparisons of relative levels of specific antigens were done by quantitative densitometry using Image J Software.

### Histology and Immunohistochemistry

Lenses were dissected from K6W and Wt mice. They were embedded in OCT, frozen and subsequently sectioned. Lens cryosections or cells growing on coverslips were fixed with 4% parafomaldehyde, permeabilized, blocked and incubated with antibodies using standard protocols. To assess proliferation in embryonic mouse lens sections wildtype and transgenic pregnant female mice were intraperitoneally injected with BrdU 2 h before euthanization.

### Antibodies

Antibodies to Ub were produced in this laboratory [44]. Antibodies to lamin A/C, Brdu, phosphor-H3, lamin A/C (phospho 392) and PDI were purchased from Abcam (Cambridge, MA). Antibodies to actin and p27^KIP^ were purchased from Santa Cruz Biotechnology (Santa Cruz, CA). The antibodies to RGS(His)_4_ and oligomer were purchased from Qiagen (Valencia, CA) and Invitrogen (Carlsbad, CA), respectively. Antibodies to DNAse IIβ and αA-crystallin were kind gifts from S. Nagata (Kyoto University, Yoshida, Sakyo-ku, Kyoto) and Nicolette Lubsen (University of Nijmegen, Netherlands), respectively. Secondary anti-mouse horseradish peroxidase and Texas Red-conjugated and anti-rabbit HRP and FITC-conjugated antibodies were from Jackson ImmunoResearch Laboratories (West Grove, PA).

### CDK1/Cyclin B Activity Assay

The CDK1/cyclin B *in vitro* kinase assay was performed using an HTScan CDK1/CycB Kinase Assay Kit (Cell Signaling), as per manufacturer's instructions. To assess inhibition of Cdk1 activity by recombinant p27^kip1^ protein (purified in this laboratory), p27^kip1^ was added at different concentrations to assay mixture.

### 2 Dimensional Electrophoresis

Lens proteins were analyzed by two-dimensional electrophoresis as described previously [45]. Spots of interest were identified by mass spectrometry-based protein sequencing [27].

## Supporting Information

Figure S1 Characterization of K6W-Ub using NMR, biochemical and cell expression. (A) Amide chemical shift perturbations (CSP), K6W versus WT ubiquitin, as a function of residue number. Residue 6 is indicated by an asterisk. The horizontal bars on the top indicate elements of the secondary structure in ubiquitin. (B) Head-on photograph of 1-month old mouse lens. Lens from animals expressing Wt-Ub are clear comparable to wild type. (C) Fluorescent micrographs show that HLE cells that express K6W-Ub accumulate protein aggregates (green) that colocalize with ubiquitin (red). (D) Fluorescent micrographs show that HLE cells that express K6W-Ub accumulate perinuclear actin aggregates (red). Immunohistochemistry was used to localize protein aggregates, ubiquitin and actin using anti-oligomer, anti-ubiquitin and anti-beta actin antibodies respectively. DAPI was used to stain nuclei. (E) Densitometric quantification of the deubiquitination assay shows that conjugates formed by K6W-Ub are as readily dismantled as those formed with Wt-Ub.
(0.33 MB TIF)

Figure S2 K6 on Ub is required to direct lens proliferation and differentiation. (A, B) Fluorescent micrographs of E18.5 K6W-Ub-expressing and Wt lenses show distribution of connexin 43, an epithelial lens cell marker. Wt lenses show an organized monolayer of cells. While, transgenic lenses show a multilayered epithelium consisting of disorganized lens epithelial cells. DAPI was used to stain nuclei. (C–F) Electron micrographs of E18.5 K6W-Ub lenses at the junction of epithelial cells and fiber cell. (C) The Wt lens shows a single layered epithelium, whereas the K6W-Ub expressing lens epithelium is thick composed of multiple layers and disorganized cells. In addition, transgenic lenses (F) show accumulation of cellular debris, vacuoles and disorganized cell structure when compared to wild type (E).
(1.05 MB TIF)

Figure S3 K6 on Ub is required to direct lens differentiation. (A, B) Fluorescent micrographs of P2 K6W-Ub lenses show retained endoplasmic reticulum (green) by the presence of protein disulfide isomerase in the OFZ of the lens when compared to wild type. Immunohistochemistry was used to detect protein disulfide isomerase, using anti-PDI antibodies. (G, H) Electron micrographs of E18.5 K6W-Ub lenses show retention of mitochondria in fiber cells from the nascent core of the lens when compared to wild type.
(0.61 MB TIF)

Figure S4 Expression of K6W-Ub diminishes with time. (A) Light micrographs of 4-month mice Wt and K6W-Ub-expressing mice. Right panel. Lenses expressing K6W-Ub retain nuclei in the core of the lens (insert) whereas the core of Wt lenses are free of nuclei, left side. (B) Western blot for K6W-Ub in E18.5, P1, P3, P6 and P45 lenses. Levels of K6W-Ub in transgenic lenses at E18.5, P1 and P3 are high, but, as the lens ages to P45, relative levels of the transgene decrease. Lenses from wildtype and transgenic animals were lysed and expression of transgene was determined by western blotting using anti-RGS(His)4. (C) Real time PCR results shows that expression of K6W-Ub (transgene) relative to GAPDH at the transcriptional level (mRNA) does not change with age. RNA was extracted from lenses of animals that express K6W-ub at ages P1, P6 and P30. Twelve lenses from different animals were used per age group.
(0.65 MB TIF)
