# Supplementary figures and images for: Perturbing the Ubiquitin Pathway Reveals How Mitosis Is Hijacked to Denucleate and Regulate Cell Proliferation and Differentiation In Vivo
Source: PLoS One. 2010 Oct 20;5(10):e13331. doi: 10.1371/journal.pone.0013331 (PMC2958118; doi:10.1371/journal.pone.0013331)

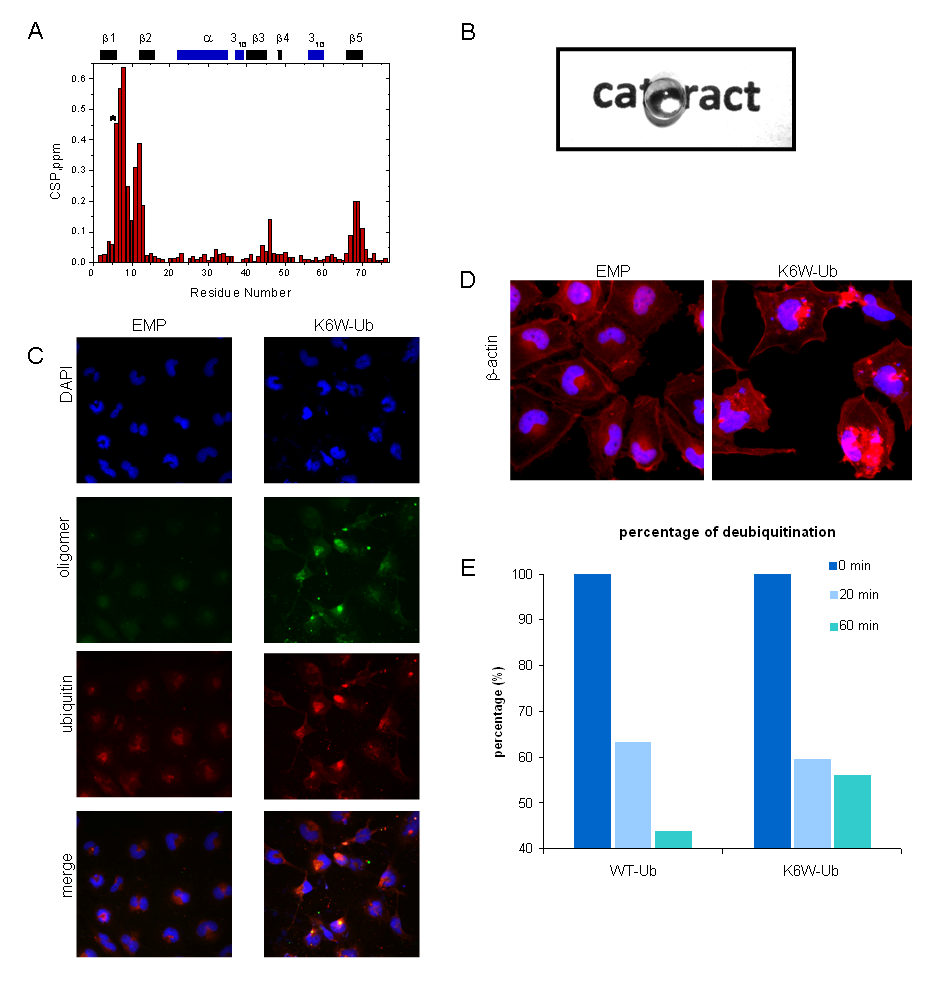

Supplement: Figure S1 — Characterization of K6W-Ub using NMR, biochemical and cell expression. (A) Amide chemical shift perturbations (CSP), K6W versus WT ubiquitin, as a function of residue number. Residue 6 is indicated by an asterisk. The horizontal bars on the top indicate elements of the secondary structure in ubiquitin. (B) Head-on photograph of 1-month old mouse lens. Lens from animals expressing Wt-Ub are clear comparable to wild type. (C) Fluorescent micrographs show that HLE cells that express K6W-Ub accumulate protein aggregates (green) that colocalize with ubiquitin (red). (D) Fluorescent micrographs show that HLE cells that express K6W-Ub accumulate perinuclear actin aggregates (red). Immunohistochemistry was used to localize protein aggregates, ubiquitin and actin using anti-oligomer, anti-ubiquitin and anti-beta actin antibodies respectively. DAPI was used to stain nuclei. (E) Densitometric quantification of the deubiquitination assay shows that conjugates formed by K6W-Ub are as readily dismantled as those formed with Wt-Ub. (0.33 MB TIF) [file pone.0013331.s001.tif]

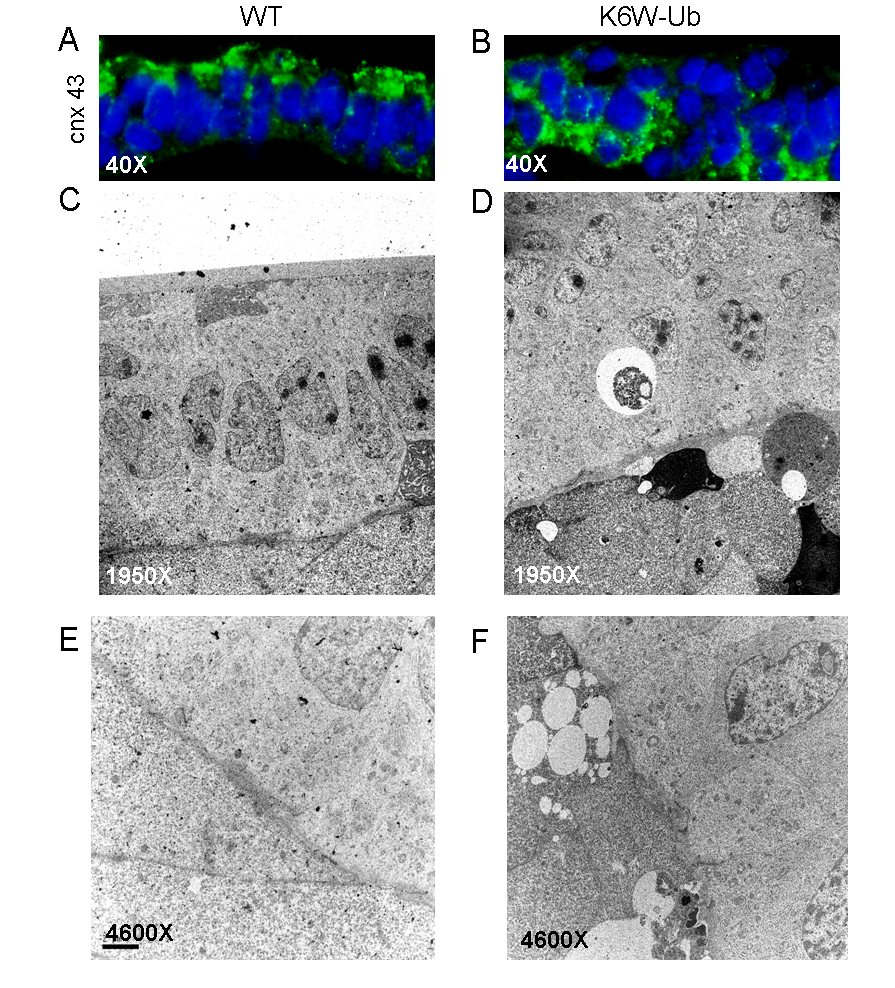

Supplement: Figure S2 — K6 on Ub is required to direct lens proliferation and differentiation. (A, B) Fluorescent micrographs of E18.5 K6W-Ub-expressing and Wt lenses show distribution of connexin 43, an epithelial lens cell marker. Wt lenses show an organized monolayer of cells. While, transgenic lenses show a multilayered epithelium consisting of disorganized lens epithelial cells. DAPI was used to stain nuclei. (C–F) Electron micrographs of E18.5 K6W-Ub lenses at the junction of epithelial cells and fiber cell. (C) The Wt lens shows a single layered epithelium, whereas the K6W-Ub expressing lens epithelium is thick composed of multiple layers and disorganized cells. In addition, transgenic lenses (F) show accumulation of cellular debris, vacuoles and disorganized cell structure when compared to wild type (E). (1.05 MB TIF) [file pone.0013331.s002.tif]

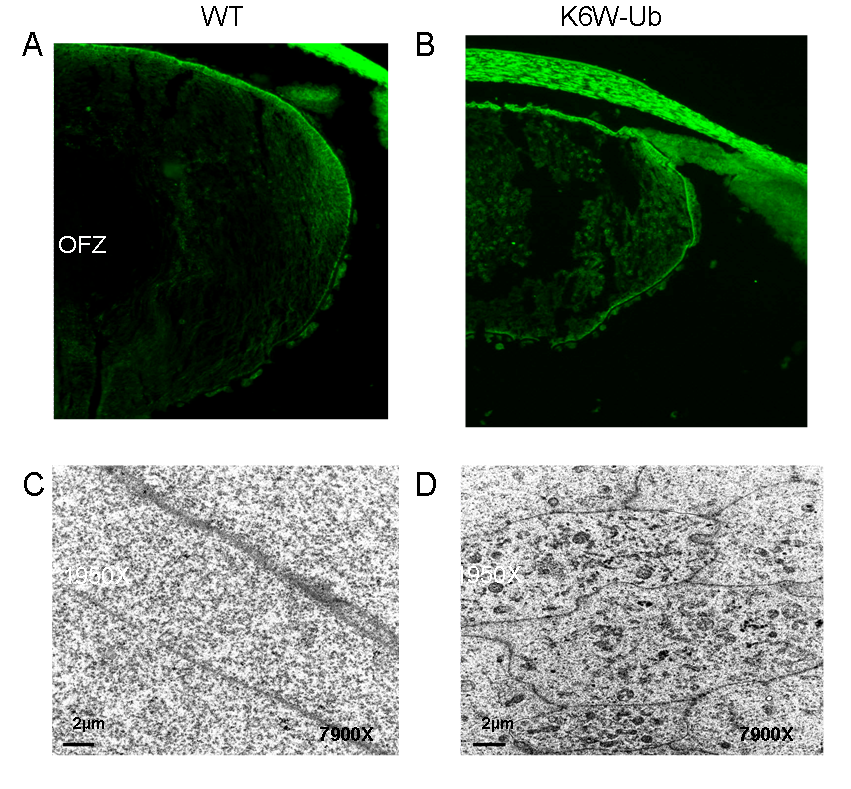

Supplement: Figure S3 — K6 on Ub is required to direct lens differentiation. (A, B) Fluorescent micrographs of P2 K6W-Ub lenses show retained endoplasmic reticulum (green) by the presence of protein disulfide isomerase in the OFZ of the lens when compared to wild type. Immunohistochemistry was used to detect protein disulfide isomerase, using anti-PDI antibodies. (G, H) Electron micrographs of E18.5 K6W-Ub lenses show retention of mitochondria in fiber cells from the nascent core of the lens when compared to wild type. (0.61 MB TIF) [file pone.0013331.s003.tif]

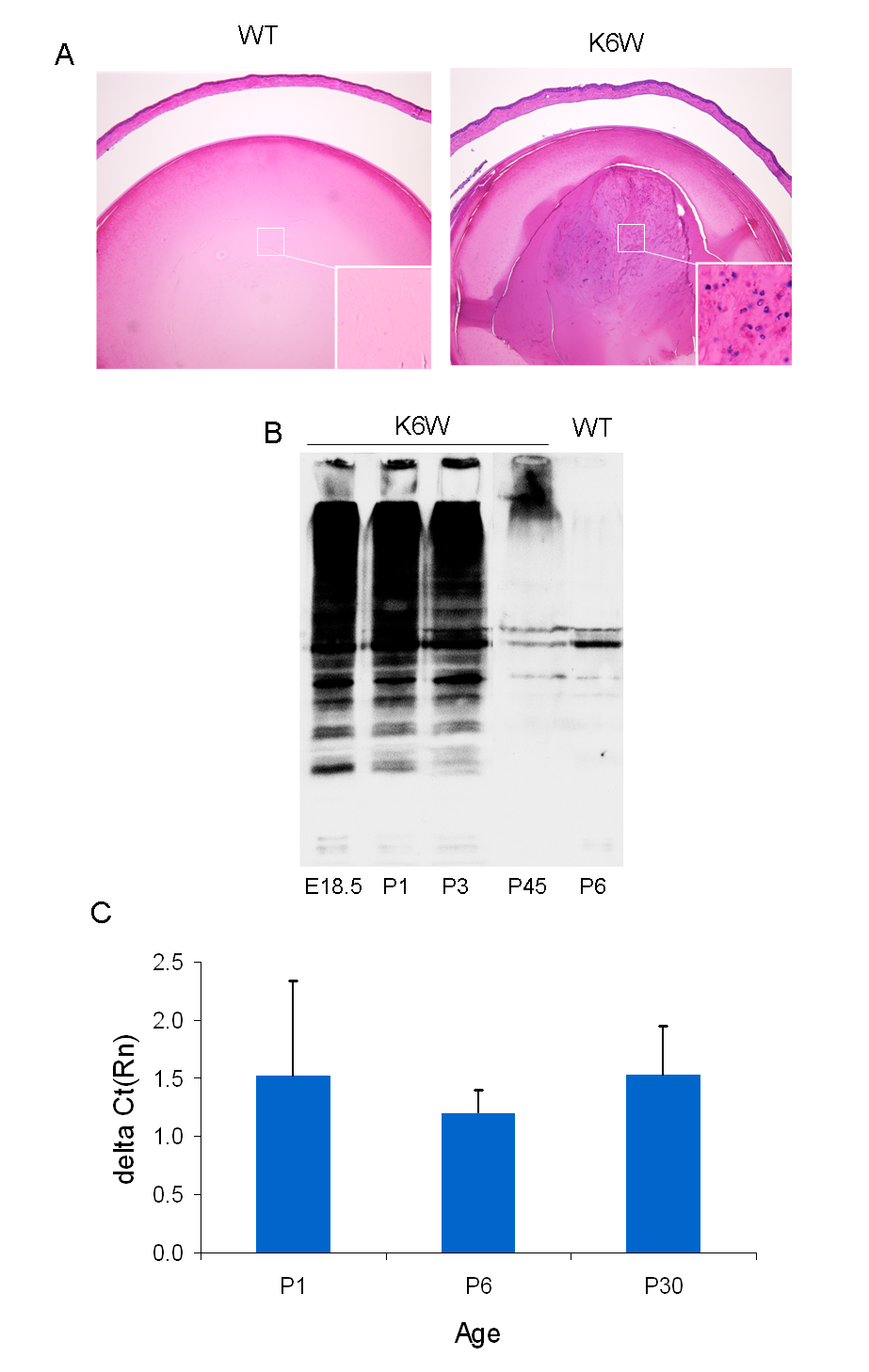

Supplement: Figure S4 — Expression of K6W-Ub diminishes with time. (A) Light micrographs of 4-month mice Wt and K6W-Ub-expressing mice. Right panel. Lenses expressing K6W-Ub retain nuclei in the core of the lens (insert) whereas the core of Wt lenses are free of nuclei, left side. (B) Western blot for K6W-Ub in E18.5, P1, P3, P6 and P45 lenses. Levels of K6W-Ub in transgenic lenses at E18.5, P1 and P3 are high, but, as the lens ages to P45, relative levels of the transgene decrease. Lenses from wildtype and transgenic animals were lysed and expression of transgene was determined by western blotting using anti-RGS(His)4. (C) Real time PCR results shows that expression of K6W-Ub (transgene) relative to GAPDH at the transcriptional level (mRNA) does not change with age. RNA was extracted from lenses of animals that express K6W-ub at ages P1, P6 and P30. Twelve lenses from different animals were used per age group. (0.65 MB TIF) [file pone.0013331.s004.tif]
